# Outcomes of Multi-Drug Resistant Tuberculosis (MDR-TB) among a Cohort of South African Patients with High HIV Prevalence

**DOI:** 10.1371/journal.pone.0020436

**Published:** 2011-07-22

**Authors:** Jason E. Farley, Malathi Ram, William Pan, Stacie Waldman, Gail H. Cassell, Richard E. Chaisson, Karin Weyer, Joey Lancaster, Martie Van der Walt

**Affiliations:** 1 Johns Hopkins University School of Nursing, Baltimore, Maryland, United States of America; 2 Bloomberg School of Public Health, Baltimore, Maryland, United States of America; 3 School of Medicine, Baltimore, Maryland, United States of America; 4 Center for Tuberculosis Research, Baltimore, Maryland, United States of America; 5 Eli Lilly and Company, Indianapolis, Indiana, United States of America; 6 South African Medical Research Council, Tuberculosis Epidemiology and Intervention Research Unit, Pretoria, South Africa; National Institute for Infectious Diseases (L. Spallanzani), Italy

## Abstract

**Background:**

Multidrug-resistant tuberculosis (MDR-TB) is a major clinical challenge, particularly in patients with human immunodeficiency virus (HIV) co-infection. MDR-TB treatment is increasingly available, but outcomes have not been well characterized. South Africa has provided MDR-TB treatment for a decade, and we evaluated outcomes by HIV status for patients enrolled between 2000 and 2004 prior to anti-retroviral access.

**Methods:**

We assessed treatment outcomes in a prospective cohort of patients with MDR-TB from eight provincial programs providing second line drugs. World Health Organization definitions were used. Results were stratified by HIV status.

**Results:**

Seven hundred fifty seven patients with known HIV status were included in the final analysis, and HIV infection was documented in 287 (38%). Overall, 348 patients (46.0%) were successfully treated, 74 (9.8%) failed therapy, 177 (23.4%) died and 158 (20.9%) defaulted. Patients with HIV were slightly younger and less likely to be male compared to HIV negative patients. Patients with HIV were less likely to have a successful treatment outcome (40.0 vs. 49.6; P<0.05) and more likely to die (35.2 vs. 16.2; P<0.0001). In a competing risk survival analysis, patients with HIV had a higher hazard of death (HR: 2.33, P<0.0001). Low baseline weight (less than 45 kg and less than 60 kg) was also associated with a higher hazard of death (HR: 2.52, P<0.0001; and HR: 1.50, P<0.0001, respectively, compared to weight greater than 60 kg). Weight less than 45 kg had higher risk of failure (HR: 3.58, P<0.01). Any change in treatment regimen was associated with a higher hazard of default (HR: 2.86; 95% CI 1.55–5.29, P<0.001) and a lower hazard of death (HR: 0.63, P<0.05).

**Conclusions:**

In this MDR-TB treatment program patients with HIV infection and low weight had higher hazards of death. Overall treatment outcomes were poor. Efforts to improve treatment for MDR-TB are urgently needed.

## Introduction

Drug-resistant tuberculosis (TB) remains a growing threat to public health despite advances made in treatment and diagnosis over the past decade [1], [2], [3], [4]. TB strains resistant to the first-line drugs isoniazid and rifampin, called multidrug-resistant TB (MDR-TB), now account for 5% of all TB cases globally. Extensively drug-resistant (XDR) TB, has been reported from more than 58 countries and is estimated to occur in up to 10% of MDR-TB patients [5]. Treatment of MDR-TB remains challenging and complex, and treatment success is considerably lower than drug-susceptible TB [6]. Sub-Saharan Africa is especially burdened with drug-resistant TB. South Africa ranks fourth among all countries for TB incidence [4] and TB remains the leading cause of mortality in HIV-infected patients; South Africa has the highest number of TB deaths attributable to HIV, at 53% [7], [8], [9], [10], [11].

The high prevalence of MDR and XDR-TB in South Africa underscores the importance of effective treatment programs for drug-resistant TB [4]. HIV co-infection complicates TB therapy and is associated with delays in diagnosis and poorer treatment outcomes [12], [13]. Expanding access to MDR-TB therapy is urgently needed, yet poor implementation of such therapy can worsen the problem of XDR-TB. Understanding risk factors for poor treatment outcomes among MDR-TB patients is necessary to improve treatment outcomes [5], [14]. Further, outcomes of MDR-TB treatment and the impact of HIV on treatment outcomes are not well described in South Africa. Further, while recent studies have shown the benefit of anti-retroviral therapy during TB treatment, integration of TB and HIV care services remains difficult in many areas of South Africa [15]. We therefore examined a large prospective cohort with a high prevalence of HIV across South Africa who received a standardized second-line therapy and programmatic management for MDR-TB to determine overall treatment outcomes among patients with and without HIV.

## Methods

### Ethics Statement

The study was approved by the Ethics Committee at the South African Medical Research Council, eight provincial level research committees, and the Institutional Review Board of The Johns Hopkins University Bloomberg School of Public Health.

### Patients and procedures

Adults aged 18 and older who presented with at least one culture confirmed bacteriological diagnosis of MDR-TB at one of ten participating MDR-TB treatment centers from eight South African provinces between 2000 and 2004 were prospectively enrolled in the study. Previously treated MDR-TB cases were excluded due to increased potential for second-line drug (SLD) resistance and limited access of centers to SLD resistance testing. MDR-TB was defined as growth of *M. tuberculosis* from sputum or another specimen with resistance minimally to isoniazid (MIC cutpoint, 0.2 and 1 mg/l) and rifampin (MIC cutpoint, 40 mg/l). Culture and susceptibility testing for all first line anti-tuberculosis drugs was performed at National Health Laboratory Service (NHLS) certified labs with an extensive internal quality assurance program associated with each treatment center. Written informed consent was obtained from each study participant before enrollment in the cohort.

Since 2000, all MDR-TB centers in South Africa have used standardized programmatic management of MDR-TB (DOTS-Plus), following a uniform approach to patient management and treatment. The program implemented standardized recording and reporting case record forms, completed by physicians and nurses. All treatment and other management decisions were made according to the DOTS-Plus protocol and by the treating team. The study team was available for clarification of study related documentation throughout the study, but otherwise was not involved in completion of the case record forms. All case record were collected by the research team from the site and manually entered into an Access database. A standardized second-line treatment regimen was used, which included a 4 to 6 month hospital-based intensive phase of pyrazinamide, ethambutol, ethionamide, ofloxacin, and either amikacin or kanamycin. This was followed by an additional 12 to 18 months of the same regimen omitting the injectable agent and pyrazinamide in the continuation phase. Treatment duration was based on when culture conversion took place; therefore, if culture conversion occurred more quickly the clinician could shorten the treatment duration at his/her discretion. The protocol specified obtaining a repeat culture and first line DST on hospital admission to confirm MDR-TB. For patients who did not have baseline culture positivity at enrollment (i.e. contaminated specimen or specimen results not available), the clinician's discretion determined if MDR-TB treatment was initiated and the appropriate duration. Time from diagnosis (i.e. date of the positive culture result at the initial outpatient evaluation) to treatment initiation was recorded. Weight-based dosing was used, and ethambutol was replaced with terizidone or cycloserine when resistance was identified. HIV testing was offered and information on previous HIV diagnoses was obtained from participants. Antiretroviral therapy was only available for TB patients in late 2004 and therefore had no impact on MDR-TB cases within this cohort.

Patients were seen at regular intervals during the intensive phase and at least monthly during the continuation phase. Any change in treatment regimen (i.e. changes in dosing of a drug, changes in the frequency of administration or discontinuation) throughout the intensive or continuation phase of treatment was recorded. The treating team assessed outcomes using criteria contained in the study protocol and recorded data on case report forms. We assessed all treatment outcomes as described in Laserson et al. (2005) after completion of the study and assigned treatment outcomes accordingly [16]. Any patients classified as treatment failure underwent a second review for additional verification of clinical outcome. Discrepancy was rare, but was resolved by a discussion between members of the research team. In all circumstances, consensus was reached and a final decision was made by the two reviewers.

### Statistical analysis

To compare treatment outcomes, we combined Completion and Cure into a single category and defined four mutually exclusive outcomes: Completion/Cure, Failure, Default, and Death. Demographic and clinical data were compared using t-tests, chi-square and Fisher's exact test as appropriate. Differences in treatment outcomes among patients were evaluated by HIV infection status. Time to treatment outcomes (from treatment initiation to each outcome) was also examined by comparing their cumulative incidence in the presence of competing risks following methods by Gooley et al. [17] using the SAS macro COMPRISK [18]. In the presence of multiple competing events, the cumulative incidence, defined as the probability of observing a particular cause of an event for an individual given a set of characteristics, is preferred over the traditional Kaplan-Meier (KM) estimate since KM estimates are known to be bias when events are dependent (i.e., KM estimates assume events are independent and censors the competing events). Cumulative incidence estimates were computedby HIV status, and baseline weight group.

To examine multivariate factors associated with time to death, default or failure, and to test modification of factors by HIV status, a competing risks regression was conducted to compute cause-specific relative hazards using the Cox proportional hazards model. Hazard ratios and their 95% Confidence Intervals (CI) are reported with ratios greater than 1 indicating more rapid time to a particular event and ratios under 1 indicating slower time to an event. Interactions with HIV status were tested for all covariates and evaluated using the likelihood ratio statistic. All analyses were conducted in SAS V9.2 (SAS Institute Inc., Cary, NC, USA).

## Results

### Baseline characteristics

We enrolled 1,023 patients in the cohort between 2000 and 2004. Among these 757 (74%) patients had a known HIV test result which included 448 (59%) men. The overall mean age for the entire cohort was 36.5 years (range 18–75). The overall mean age for women was 33.2 years (10.4, standard deviation; S.D.), compared to 38.8 years (10.5, S.D.) for men (P<.0001). Thirty-eight percent of patients (n = 287) had HIV infection and 62% (n = 470) were HIV negative. The majority of patients were previously treated for TB (92%) and almost all (98%) had pulmonary disease. Although all patients were laboratory confirmed with MDR-TB at the referring center, repeat culture for confirmation on hospital admission was completed in 556 (72%), therefore 28% were enrolled in the cohort using culture results obtained at the referring treatment facility. Two hundred ninety (28%) individuals were tested for baseline ethambutol resistance; among these, resistance was detected in 197 subjects (68%). The mean treatment duration for the cohort was 393 days (204.3, S.D.).

Baseline demographic characteristics stratified by HIV status are shown in Table 1. Patients with HIV were younger than HIV negative patients (34.8 vs. 37.6 years; P<0.001) and were less likely to be male (53.7 vs. 62.6%; P<0.05). The mean time from culture-based diagnosis of MDR-TB to initiation of treatment was > two months for both groups. The baseline weight, sputum smear status, mycobacterial culture positivity and ethambutol resistance did not differ between patients with and without HIV infection.

**Table 1:** Baseline Demographic Characteristics by HIV Status.

	Positive	Negative	p-value
	N = 287	N = 470	
	Mean (SD)	Mean (SD)	
Age (Years)	34.8 (8.8)	37.6 (11.7)	0.0007
Pre-Treatment Weight (Kg)	50.3 (11.3)	50.2 (10.4)	0.9650
	Mean (IQR)	Mean (IQR)	
Time from Diagnosis to	63.9 (46.0)	70.4 (48.5)	0.1847
Treatment (Days)∧ Median	50	54	
	n (%)	n (%)	
Males	154 (53.7)	294 (62.6)	0.0157
Pre-Treatment Smear Positive	164 (57.1)	270 (57.5)	0.9346
Pre-Treatment Culture Positive*	209 (72.8)	347 (73.8)	0.7607
Ethambutol Resistance	72 (72.0)	125 (65.8)	0.2815

∧ Interval between culture positive diagnosis at initial evaluation to time of treatment initiation in MDR-TB Hospital *All (100%) of patients had a positive culture on initial evaluation and referral to the study. The numbers presented here reflect repeat testing on cohort enrollment.

### Overall Treatment Outcomes

The treatment success rate was 46% (n = 348), with 21% who were cured and 25% who completed treatment. Treatment failure occurred in 74 patients (9.8%), 177 patients (23.4%) died during treatment and 158 patients (20.9%) defaulted. Patients with HIV were less likely to have a successful treatment outcome than HIV negative patients (40% vs. 49.6%, P<0.05) and were significantly more likely to die (35.2% vs. 16.2%, P<0.0001, (Table 2)). Patients with HIV had a lower treatment failure rate than HIV negative patients (4.2 vs. 13.2%, P<0.0001). Treatment duration was significantly different between HIV positive and negative patients (349 days vs. 419 days; P<0.0001).

**Table 2:** Comparison of Treatment Outcomes by HIV Status.

	HIV Status		
	Positive	Negative	p-value*
	N = 287	N = 470	
	n (%)	n (%)	
Success	115 (40.0)	233 (49.6)	<0.05
Failure	12 (4.2)	62 (13.2)	<0.0001
Default	59 (20.6)	99 (21.1)	0.87
Death	101 (35.2)	76 (16.2)	<0.0001

*Based on Chi-Square Test.

**Figure 1:**
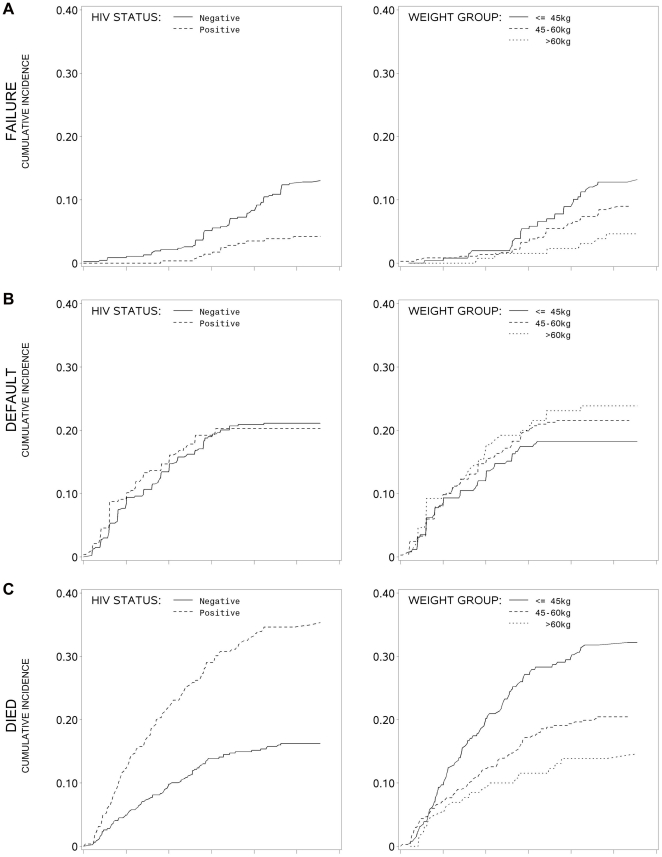
Cumulative Incidence of Treatment Outcomes by HIV status and weight group. A =  Failure; B =  Default; C =  Died.

Cumulative incidences estimates of each event (failure, default or death) by HIV status, treatment regimen, and weight group are shown in Figure 1. Although inferential comparison by strata would not be appropriate, examining the cumulative incidence it is evident that patients with HIV had a greater probability of earlier death, a slightly higher probability of earlier failure and no differences in probability of default. Death occurs substantially earlier for persons less than 45 kgs with limited differences in failure and default by weight group.

Our competing risk analysis (Table 3) shows that males have a lower hazard ratio for failure (HR 0.67; 95% CI 0.46–0.98, P<.05), but no sex differences were noted in default or death. HIV infection is associated with a significantly increased hazard of death (HR: 2.52; 95% CI 2.04–3.13, P<.0001) and decreased hazard of failure (HR: 0.41; 95% CI 0.17–0.96, P<.05); however, HIV infection was not associated with default. The lowest weight group at baseline (<45 kg) had a higher hazard of death (HR: 2.56; 95% CI 2.06–3.18, P<.0001) and failure (HR: 3.61; 95% CI 1.53–8.53, P<.01). The intermediate weight group at baseline (46–60 kgs) also had a higher hazard of death (HR: 1.48; 95% CI 1.26–1.74, P<.0001) and failure, although time to failure was not statistically significant at the 0.05 level. No differences were noted between time of diagnosis to treatment initiation for any outcome. The hazard ratios by treatment outcome are depicted graphically (Figure 1) for ease of interpretation. After completing this analysis, interactions of HIV with all co-variates were tested to determine modification of co-variates by HIV status; no significant interactions with HIV were found.

**Table 3:** Competing Risk Model of Failure, Default and Death.

		Failure				Default				Death			
		Hazard Ratio	95% CI		p-value	Hazard Ratio	95% CI		p-value	Hazard Ratio	95% CI		p-value
**Age (years, centered @ 36)**		1.00	0.97	1.02	0.772	1.00	0.98	1.01	0.467	1.00	0.99	1.01	0.899
**Sex**	Males	**0.67**	**0.46**	**0.98**	**0.041**	0.88	0.51	1.54	0.659	1.12	0.87	1.43	0.377
**HIV Status**	Positive	**0.41**	**0.17**	**0.96**	**0.040**	1.07	0.74	1.56	0.721	**2.52**	**2.04**	**3.13**	**<.0001**
**DX TX Group (ref <30 days):**	30-60 days	1.01	0.55	1.87	0.968	1.06	0.66	1.68	0.821	1.08	0.77	1.52	0.664
	>60 days	1.34	0.83	2.17	0.235	1.02	0.52	2.00	0.965	0.86	0.49	1.50	0.591
**Weight Group (ref >60 kg):**	≤45 kg	**3.61**	**1.53**	**8.53**	**0.003**	0.83	0.43	1.58	0.567	**2.56**	**2.06**	**3.18**	**<.0001**
	45-60 kg	2.28	0.87	6.02	0.095	0.96	0.64	1.44	0.841	**1.48**	**1.26**	**1.74**	**<.0001**

AIC =  4854.266.

-2 Log L =  4812.266.

Test of Weight Group 1 vs 2: Wald = 3.5642 p = 0.0590 Wald = 0.5475 p = 0.4593 Wald = 17.6184.

Test of DxTx* Group 1 vs 2: Wald = 2.9411 p = 0.0863 Wald = 0.0155 p = 0.9011 Wald = 0.9187.

*DxTx is the time from culture diagnosis to treatment initiation.

## Discussion

This study demonstrates poor treatment outcomes from a large DOTS-Plus cohort in sub-Saharan Africa prior to the availability of ART. Using a standardized regimen and treatment protocol, the overall mortality rate of 23.4% is markedly higher than mortality among MDR-TB treatment cohorts from other countries [6], [19] with similar epidemiologic characteristics. Mortality was twice as high among HIV co-infected patients as patients without HIV with a substantial probability for earlier mortality which is consistent with the findings of other investigators [20]. This finding explains the differences noted in treatment duration between the two groups. Further, it is also important to note that mortality among patients without HIV infection was greater than seen by other investigators [6]. Studies on the impact of ART in the region have demonstrated both a reduction in TB incidence [21] along with a survival benefit of persons receiving earlier initiation of ART in patients with drug susceptible TB [22]. Access to ART would likely have resulted in improved survival among this cohort, though the impact on treatment success cannot be estimated. Increasing access to early ART is an urgent priority to addressing the severity of the MDR-TB/HIV epidemic [15], [23].

This analysis demonstrates striking differences in risk of death and failure when stratified by baseline weight and controlling for other baseline factors. This finding is similar to other cohorts studies throughout the world who also demonstrate a direct relationship to low body weight and poor MDR-TB treatment outcomes [24], [25]. Little is known about the impact of weight on adverse drug reactions or pharmacodynamics. We believe this is an important area for continued investigation, particularly correlations of low body weight with both adverse drug reactions and therapeutic drug levels of MDR-TB treatments. In light of our data demonstrating a substantial treatment delay between establishing the diagnosis to treatment initiation, we believe decentralized care models and more rapid diagnostic with improved turnaround time may be appropriate interventions to improve this issue and further study is needed.

The overall success rate of 46% is lower than reports from other cohorts of MDR-TB patients [6], [7], [26]; however, differences in HIV prevalence, treatment regimen and duration are important considerations in making these comparisons. Another recently published cohort of MDR-TB treatment outcomes obtained from a single center in KwaZulu-Natal (2000 – 2003) found successful treatment outcomes of 43%, similar to our findings [27]. This is an important comparison, as our study does not include data from KwaZulu-Natal. Comparing these studies, it is clear that treatment success is poor across the entire country. A systematic review by Orenstein and colleagues documented an overall treatment success rate of 62% (95% CI 58%–67%) among 33 studies from around the world. The combination of a treatment length greater than 18 months and use of directly observed therapy (DOT) throughout treatment were associated with treatment success [6]. Although not statistically significant, it was noted that individualized treatment regimens offered a trend toward greater success (64% vs. 54%, P = 0.08) than a standardized approach. This study, however, included only two of the 22 high TB burden countries and no high TB/HIV burden countries [6]; however, another meta-analysis by Johnston et al, which included slightly more high burden countries including South Africa, found a similar 62% success rate [2]. In comparison, our study findings demonstrate poor outcomes in a program using a shorter regimen duration and a standardized approach. The clinicians treating our patients did not have access to SLD susceptibility testing and ethambutol resistance was identified among 68% of patients whose isolate was tested; it is possible that many patients received fewer than 4 active drugs which could limit treatment efficacy [2]. One systems level issue in South Africa that may contribute to poorer outcomes is the time from MDR-TB diagnosis to treatment initiation. We found an average diagnosis to treatment initiation delay of more than 2 months. We believe there are many factors that contribute to this delay. First, a delay could be present in the notification of the culture result from the laboratory; second, delays may have been encountered in notifying the patient of the result and referring him/her to the MDR-TB treatment facility; third, the availability of an inpatient bed in an MDR-TB facility may have resulted in additional delay. Finally, patients in our study received DOT during the intensive phase of therapy, but many had self-administered therapy during the continuation phase and this could have contributed to poorer outcomes. We do note that the majority of our cohort (92%) was re-treatment cases. Although we did not evaluate this, a possible cause for this high rate of acquired MDR-TB among this group is that the DOT infrastructure requires strengthening at all levels of the TB program.

Treatment failure among this cohort approached 10%. This is greater than rates in cohorts in settings with low HIV prevalence (<5%) where failure rates range between 0% and 4% among new MDR-TB patients [26]. We do not have second line drug susceptibility results for our patients as yet, though testing is ongoing, and we cannot therefore determine whether individuals who failed treatment or died had XDR-TB either at baseline or acquired during MDR treatment. In South Africa, initial and acquired XDR-TB (re-infection during treatment) are both likely to have been present and may have contributed to the poor outcomes observed [28]. One particular treatment approach that may remedy the problems associated with exogenous re-infection from limited infection control in these settings is to pursue community-based MDR-TB treatment. A recent evaluation within KwaZulu-Natal demonstrated a decreased time to treatment initiation along with a shortened time to culture conversion in patients treated in the community [29]. In this analysis, men had a lower hazard ratio for failure. Overall, we did not find that they were less likely to fail than women, but simply they did so less rapidly. We speculate this may have been related to pre-treatment health status (i.e. earlier presentation resulting in lower acuity), but data beyond baseline weight and time from diagnosis to treatment initiation was not available.

Although HIV infection was not associated with default in this study, we did find a high frequency of default (just over 20%) among both HIV positive and negative subjects. Other investigators have found similar findings in South Africa. An analysis in the West Coast/Winelands found an overall default rate of 29% among MDR-TB patients [30], although differences between HIV positive and negative patients was not evaluated. While patients who defaulted were not tracked in this study to determine the reasons for default, we never the less believe this is a complex issue. Evaluations of MDR-TB treatment default in South Africa have determined that healthcare worker attitude and substance abuse to be associated with patient default [31]. We believe in our cohort many issues, both programmatic and treatment related, lead to default. Our experience tells us that many patients are not informed on referral to MDR-TB treatment centers about the duration of therapy or, more specifically, the duration of inpatient hospitalization. Further, adverse drug reactions are commonly reported in the literature. Our analysis of this situation is currently ongoing. Changes are underway by the South Africa Department of Health to address challenges in the treatment paradigm including community-based treatment models [32] that will likely help to reduce the high rate of default experienced in this and other MDR-TB cohorts.

As with any large clinical cohort study, our study has several limitations. First, although a standard protocol and data collection format were used, data on HIV status and CD4 counts are not uniformly available. It is well known that TB patients in South Africa have an exceptionally high HIV prevalence and it is believed that MDR-TB patients have similar rates [9]. Our current finding of 38% likely under represent the true prevalence of HIV as a large proportion of the original cohort were not tested, skewing results for those with documented HIV infection, who may have been more ill. Our study excluded retreatment cases as we did not have access to SLD resistance testing which may limit generalizabiliy to programs that provide this standard MDR-TB regimen to retreatment cases. Finally, data on adherence to treatment was poorly documented, particularly during the continuation phase of treatment. Despite these limitations, we believe this study provides an important evaluation of treatment outcomes in the absence of ART.

### Summary

This study describes treatment outcomes from a large prospective cohort with high HIV prevalence using a standardized MDR-TB regimen in South Africa pre-ART. Our findings emphasize the need for greater attention to program performance and other interventions to reduce the substantial mortality associated with drug-resistant tuberculosis observed here in the absence of ART. Strengthening programs by intensely evaluating treatment regimens, ensuring all HIV positive patients have access to ART, adequate staffing to support true DOT, aggressive and pro-active management of adverse drug effects, and infection control measures to prevent transmission of MDR and XDR-TB between patients and health care workers are all essential interventions [28]. Community-based treatment of MDR-TB, with sufficient staffing to assure adherence, is likely to improve treatment outcomes and will enhance the quality of life for those suffering from this illness. Integration of TB and HIV care, with increased access to ART for HIV infected MDR-TB patients, is also essential, as demonstrated by recent studies showing improved survival when ART is started during TB therapy [33].

## References

[1] Ahmad S, Al-Mutairi NM, Mokaddas E (2009). Comparison of performance of two DNA line probe assays for rapid detection of multidrug-resistant isolates of Mycobacterium tuberculosis.. Indian J Exp Biol.

[2] Johnston JC, Shahidi NC, Sadatsafavi M, Fitzgerald JM (2009). Treatment outcomes of multidrug-resistant tuberculosis: a systematic review and meta-analysis.. PLoS One.

[3] Shah NS, Moodley P, Babaria P, Moodley S, Ramtahal M (2011). Rapid diagnosis of tuberculosis and multidrug resistance by the microscopic-observation drug-susceptibility assay.. Am J Respir Crit Care Med.

[4] World Health Organization (2010). Multidrug and extensively drug-resistant TB (M/XDR-TB): 2010 Global Report on Surveillance and Response..

[5] World Health Organization (2008). Tuberculosis MDR-TB & XDR-TB: The 2008 Report..

[6] Orenstein EW, Basu S, Shah NS, Andrews JR, Friedland GH (2009). Treatment outcomes among patients with multidrug-resistant tuberculosis: systematic review and meta-analysis.. Lancet Infect Dis.

[7] Cox HS, Kalon S, Allamuratova S, Sizaire V, Tigay ZN (2007). Multidrug-resistant tuberculosis treatment outcomes in Karakalpakstan, Uzbekistan: treatment complexity and XDR-TB among treatment failures.. PLoS One.

[8] Gupta R, Kim JY, Espinal MA, Caudron JM, Pecoul B (2001). Public health. Responding to market failures in tuberculosis control.. Science.

[9] Mukadi YD, Maher D, Harries A (2001). Tuberculosis case fatality rates in high HIV prevalence populations in sub-Saharan Africa.. AIDS.

[10] Resch SC, Salomon JA, Murray M, Weinstein MC (2006). Cost-effectiveness of treating multidrug-resistant tuberculosis.. PLoS Med.

[11] World Health Organization (2008). Anti-tuberculosis drug resistance in the world: Fourth global report..

[12] Friedland G (2007). Tuberculosis, drug resistance, and HIV/AIDS: a triple threat.. Curr Infect Dis Rep.

[13] Wells CD, Cegielski JP, Nelson LJ, Laserson KF, Holtz TH (2007). HIV infection and multidrug-resistant tuberculosis: the perfect storm.. J Infect Dis.

[14] Gandhi NR, Moll A, Sturm AW, Pawinski R, Govender T (2006). Extensively drug-resistant tuberculosis as a cause of death in patients co-infected with tuberculosis and HIV in a rural area of South Africa.. Lancet.

[15] Perumal R, Padayatchi N, Stiefvater E (2009). The whole is greater than the sum of the parts: recognising missed opportunities for an optimal response to the rapidly maturing TB-HIV co-epidemic in South Africa.. BMC Public Health.

[16] Laserson KF, Thorpe LE, Leimane V, Weyer K, Mitnick CD (2005). Speaking the same language: treatment outcome definitions for multidrug-resistant tuberculosis.. Int J Tuberc Lung Dis.

[17] Gooley TA, Leisenring W, Crowley J, Storer BE (1999). Estimation of failure probabilities in the presence of competing risks: new representations of old estimators.. Stat Med.

[18] Bergstralh E (2011). Locally written SAS Macros..

[19] Nathanson E, Lambregts-van Weezenbeek C, Rich ML, Gupta R, Bayona J (2006). Multidrug-resistant tuberculosis management in resource-limited settings.. Emerg Infect Dis.

[20] Gandhi NR, Shah NS, Andrews JR, Vella V, Moll AP (2010). HIV coinfection in multidrug- and extensively drug-resistant tuberculosis results in high early mortality.. Am J Respir Crit Care Med.

[21] Lawn SD, Myer L, Bekker LG, Wood R (2006). Burden of tuberculosis in an antiretroviral treatment programme in sub-Saharan Africa: impact on treatment outcomes and implications for tuberculosis control.. AIDS.

[22] Abdool Karim SS, Naidoo K, Grobler A, Padayatchi N, Baxter C (2010). Timing of initiation of antiretroviral drugs during tuberculosis therapy.. N Engl J Med.

[23] Dheda K, Lampe FC, Johnson MA, Lipman MC (2004). Outcome of HIV-associated tuberculosis in the era of highly active antiretroviral therapy.. J Infect Dis.

[24] Leimane V, Riekstina V, Holtz TH, Zarovska E, Skripconoka V (2005). Clinical outcome of individualised treatment of multidrug-resistant tuberculosis in Latvia: a retrospective cohort study.. Lancet.

[25] Malla P, Kanitz EE, Akhtar M, Falzon D, Feldmann K (2009). Ambulatory-based standardized therapy for multi-drug resistant tuberculosis: experience from Nepal, 2005-2006.. PLoS One.

[26] Nathanson E, Gupta R, Huamani P, Leimane V, Pasechnikov AD (2004). Adverse events in the treatment of multidrug-resistant tuberculosis: results from the DOTS-Plus initiative.. Int J Tuberc Lung Dis.

[27] Brust JC, Gandhi NR, Carrara H, Osburn G, Padayatchi N (2010). High treatment failure and default rates for patients with multidrug-resistant tuberculosis in KwaZulu-Natal, South Africa, 2000-2003.. Int J Tuberc Lung Dis.

[28] Andrews JR, Gandhi NR, Moodley P, Shah NS, Bohlken L (2008). Exogenous reinfection as a cause of multidrug-resistant and extensively drug-resistant tuberculosis in rural South Africa.. J Infect Dis.

[29] Heller T, Lessells RJ, Wallrauch CG, Barnighausen T, Cooke GS (2010). Community-based treatment for multidrug-resistant tuberculosis in rural KwaZulu-Natal, South Africa.. Int J Tuberc Lung Dis.

[30] Shean KP, Willcox PA, Siwendu SN, Laserson KF, Gross L (2008). Treatment outcome and follow-up of multidrug-resistant tuberculosis patients, West Coast/Winelands, South Africa, 1992-2002.. Int J Tuberc Lung Dis.

[31] Holtz TH, Lancaster J, Laserson KF, Wells CD, Thorpe L (2006). Risk factors associated with default from multidrug-resistant tuberculosis treatment, South Africa, 1999-2001.. Int J Tuberc Lung Dis.

[32] Department of Health Republic of South Africa (2010). Decentralized Management of Multi-drug Resistant Tuberculosis: A Policy Framework for South Africa (Draft)..

[33] Gandhi NR, Moll AP, Lalloo U, Pawinski R, Zeller K (2009). Successful integration of tuberculosis and HIV treatment in rural South Africa: the Sizonq'oba study.. J Acquir Immune Defic Syndr.

